# Prevalence of sexually transmitted infections and immunization status among registered sex workers: A pilot study in lower Bavaria, Germany

**DOI:** 10.1371/journal.pone.0338710

**Published:** 2025-12-12

**Authors:** Fabian Standl, Lena Senger, Heribert Stich

**Affiliations:** 1 Institute for Medical Informatics, Biometry and Epidemiology (IMIBE), Essen University Medical Center, Essen, Germany; 2 TUM School of Medicine and Health, Graduate Center of Medicine and Health Figure, Technical University of Munich, Munich, Germany; 3 Institute for Medical Information Processing, Biometry and Epidemiology (IBE), Chair of Public Health and Health Services Research, Ludwig-Maximilians-Universität München, Medical Faculty, Munich, Germany; 4 Pettenkofer School of Public Health, Medical Faculty, Munich, Germany; 5 Institute for Medical Information Processing, Biometry and Epidemiology (IBE), Ludwig-Maximilians-Universität München, Medical Faculty, Munich, Germany; 6 District Office of Landshut, Department of Public Health Services, Achdorfer Weg 7, Landshut, Germany; Centers for Disease Control and Prevention, UNITED STATES OF AMERICA

## Abstract

**Background:**

Sex workers are often considered at elevated risk for sexually transmitted infections (STIs). This pilot study describes the socio‑epidemiological characteristics of registered sex workers in a rural German setting, estimates the prevalence of four STIs (HIV, hepatitis B [HBV], hepatitis C [HCV], and syphilis [lues]), compares these with the local population, and assesses HBV immunization coverage.

**Methods:**

Under §10 of the Prostitute Protection Act (ProstSchG), annual health consultations are mandatory; voluntary serologic testing is permitted under §19 of the Infection Protection Act. We conducted a retrospective observational monocentric pilot study using routine consultation records and voluntary serologic results from the Public Health Service (PHS) of Landshut (2017–2021). In total, 523 consultations were documented; 99 blood samples from 48 registered sex workers (2019–2021) were analyzed. Primary screening assays were followed by confirmatory tests when indicated. Crude point/period prevalences and 95% confidence intervals (95% CI) were calculated. HBV immunization was defined according to Standing Committee on Vaccination (STIKO) recommendations.

**Results:**

The cohort was predominantly female (n = 47; 97.9%), mean age 34.8 ± 11.2 years; 85.3% (n = 41) had a migration background (n = 27; 56.3% from Eastern EU countries). No acute HIV, HBV, or HCV infection was detected. Evidence of past HBV infection (anti‑HBc) was found in n = 7 (14.6%; 95% CI: 6.8–26.5), past HCV in n = 1 (2.1%; 95% CI: 0.2–9.3). Syphilis serology was reactive in 12.5% (n = 6), with n = 2 (4.2%; 95% CI: 0.9–12.7) meeting criteria for treatment‑requiring infection. HBV vaccine‑induced immunity was documented in 43.8%; only 29.2% achieved titers ≥100 mIU/ml. Compared with regional surveillance data, the prevalence of acute notifiable STIs among sex workers was not increased.

**Conclusions:**

In this rural setting, acute notifiable STIs were uncommon among registered sex workers, while past HBV infection and suboptimal HBV immunization were frequent. Public health efforts should prioritize HBV vaccination and syphilis prevention or treatment, and expand low‑threshold, trusted services tailored to this workforce.

## Background

Sex workers are frequently described as having higher risk of Sexually Transmitted Infections (STI) than the general population due to structural and behavioral determinants, including legal status, work conditions, substance use, client competition, and variable sexual or hygiene practices [1–9].

In Germany, the Public Health Service (PHS) provides annual health consultations mandated by §10 of the Prostitute Protection Act (ProstSchG) [10–12], with voluntary, anonymous medical examinations permitted under §19 of the Infection Protection Act (IfSG) [13]. An additional medical examination for infectious diseases was of secondary importance, additional examinations by a medical member of the PHS and blood analyses may only performed on a voluntary and anonymous basis [10–12].

Most research to date derives from large cities and heterogeneous designs focusing on selected infections [4,6,8,9,11,12,14–17], while rural regions remain underrepresented. Robust local data are needed to inform prevention, screening, and vaccination strategies tailored to registered sex workers.

This pilot study aimed to (i) describe socio‑epidemiological characteristics of registered sex workers in Lower Bavaria; (ii) estimate the prevalence of four defined STIs (HIV, HBV, HCV, syphilis); (iii) compare acute notifiable infections with regional surveillance data; and (iv) assess HBV immunization coverage according to Standing Committee on Vaccination (STIKO).

## Materials and methods

### Study design and setting

We conducted a retrospective, observational, monocentric pilot study in the City and District of Landshut, Lower Bavaria (Germany). Using archived standardized consultation forms and laboratory reports, we compiled routine data from mandatory health consultations [11,12] and results from voluntary, anonymous serologic testing [13] at the PHS of Landshut during the years 2017–2021. The health consultations were conducted in the context of confidential individual consultations on the premises of the Social Services of the District of Landshut, rephrasing standardized statistical paper forms were recorded and archived by the socio-pedagogical staff immediately after the consultations. The group of registered sex workers who also agreed to a blood test were sent to a medical member of the PHS for blood collection and the samples were subsequently sent to an accredited laboratory of the Bavarian Health and Food Safety Authority (LGL).

### Study population

We analyzed 523 consultations (July 01, 2017 to December 12, 2021). Serology was available for 48 unique individuals (January 01, 2019 to December 31, 2021) who consented to blood testing and were referred to an accredited state laboratory. All included individuals were 18 years or older, most had a binary gender, were registered sex workers, and were seen by the staff of the local PHS.

### Study area

The study area comprised a total of about 237,000 inhabitants (City of Landshut: nearly 72,000 and District of Landshut: over 165,000 inhabitants) with a predominantly rural structure [18]. In this region hosts nine licensed venues and no street‑based sex work [19,20] where registered sex workers carried out their activities.

### Biomedical laboratory analyses

Primary antigen/antibody screening assays were used with confirmatory testing for reactive results (Table 1). HBV markers included HBsAg, anti‑HBs, and anti‑HBc; HCV testing included antibody screening, confirmation, and HCV‑RNA verification (≥30 IU/ml). Syphilis screening (CMIA/EIA) was followed by TPPA, IgM immunoblot, and nontreponemal testing; treatment‑requiring infection was defined by reactive serology with lipoidal antibodies ≥1:8 or compatible clinical presentation. HBV vaccine‑induced immunity was defined according to STIKO (≥3 doses for primary series; anti‑HBs ≥ 100 mIU/ml considered long‑term protection) [21].

**Table 1:** Laboratory diagnostic immunoassays and interpretation of the results of the serum examination in the PHS of Landshut.

Infectious disease	Analysis	Unit	Method	Result
HIV	HIV combined antigen-antibody screening test	S/CO	CMIA/EIA	Negative < 1.00
Hepatitis B	HBs-Antigen	S/CO	CMIA/EIA	Negative < 1.00
	anti-HBs	mlU/ml	CMIA/EIA	Long-term protection for ≥ 100 mlU/ml
	anti-HBc	S/CO	CMIA/EIA	Negative < 1.00
Hepatitis C	Antibody screening test	S/CO	CMIA/EIA	Negative < 1.00
	Antibody confirmation test bands (C1 1 + , C2 1 + , NS3 2 + , NS4 2+)*		IB	
	HCV-RNA- Verification*	IU/ml	PCR	Detection with ≥ 30 lU/ml
Syphilis	Antibody screening test (qualitative) (Tp47, Tp17, Tp44.5)	S/CO	CMIA/EIA	Negative < 1.00
	Antibody detection (quantitative)*		TPPA	Negative < 1:80
	IgM antibody confirmation test bands*		IB	
	Lipoid antibody detection (qualitative)*		FR	
	Lipoid antibody detection (quantitative)*		FR	Negative < 1:8

*Only performed if the search test is positive

CIMA = chemiluminescence microparticle immunoassay

EIA = Enzyme Immuno Essay

IB = Immunoglobulins Blood Tests

PCR = Polymerase Chain Reaction

TPPA = Treponema Pallidum Particle Agglutination

FR = Framework Regions (-detection)

S/CO = Signal/ Cut-Off Ratio

mIU/ml = milli International Units per milliliter

IU/ml = International Units per milliliter

After completion of these laboratory analyses, the respective laboratory findings were sent in paper form by the laboratory in charge to the PHS of Landshut and stored securely on their premises. The individuals concerned were subsequently anonymously informed of the test results by telephone.

### Inclusion and exclusion cirteria

Only sex workers registered in the study area who clearly consented to blood sampling were included in the study. Minors, pregnant women and sex workers in need of acute medical treatment and with ambiguous information or invalid laboratory results were not included in the study population.

### Statistical analysis

We report absolute and relative frequencies and crude point or period prevalences with 95% confidence interval (95% CI). Regional comparisons used SurvStat@RKI 2.0 (cumulative prevalence per population) [22]. A logistic regression explored the association between citizenship (German vs non‑German) and the count of active or chronic infections (Nagelkerke R²). Given by the pilot sample size, analyses were descriptive and exploratory.

### Ethical approval and consent

Project number 22–0037 was discussed by the Ethics Committee of Ludwig-Maximilians-Universität München in accordance with faculty law and §15 of the Professional Code for Physicians in Bavaria. The ethics committee raised no objections to the study being conducted in the form presented. The registered sex workers had to appear at the PHS of Landshut for the mandatory health consultations according to §10 ProstSchG and provide their personal data. These personal data were strictly protected from access by unauthorized persons and documented in accordance with data protection regulations. The additional laboratory tests offered were voluntarily performed and could therefore be refused by the sex workers. Informed consent was obtained from all subjects. All methods were in accordance to the relevant guidelines and regulations.

## Results

### Pilot study overview diagram

**Fig 1:**
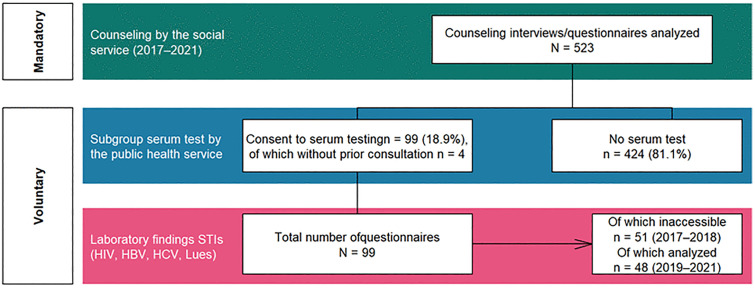
Overview diagram of the analyzed routine data in the Landshut Public Health Service area from July 1^st^ 2017 to December 31^st^ 2021.

As shown in Figure 1, between 2017 and 2021, the PHS conducted 523 consultations; 99 serologic tests from 48 individuals were performed (2019–2021). Approximately 81% (424/523) of consultations did not lead to serologic testing; 96% (95/99) of tests occurred immediately post‑consultation.

### Serum tests

#### Socio-demographic description of the serum samples.

The serology cohort had a mean age of 34.8 years (range 20–56), and was predominantly female (97.9%). A detailed breakdown of age and gender within the study group can be seen in Table 2 below.

**Table 2:** Socio-demographic characteristics and serum tests of sex workers in the PHS of Landshut, January 01, 2019 to December 31, 2021.

Years of study	Total number of serum tests (%)	Arithmetic mean of sex workers in years^*)^	Female sex workers with serum test (%)	Male sex workers with serum test (%)	Sex workers with a migration background (%)
2019	29 (100.0)	33.2	29 (100.0)	0 (0.0)	26 (89.7)
2020	11 (100.0)	35.9	10 (90.9)	1 (9.1)	10 (90.9)
2021	8 (100.0)	39.6	8 (100.0)	0 (0.0)	6 (75.0)
Total	48 (100.0)	34.8	47 (97.9)	1 (2.1)	42 (87.5)

*) one missing entry for the year 2021 (n=47).

**Fig 2:**
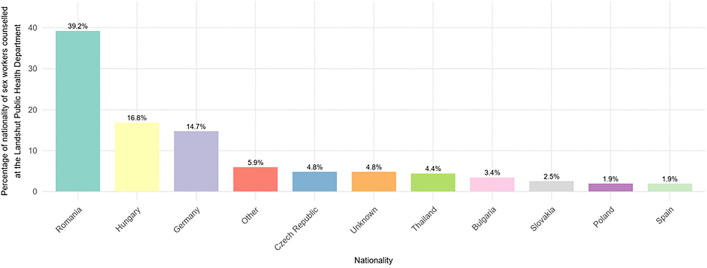
Nationality of sex workers counselled according at the Landshut Public Health Department\n (N = 523), July 1st 2017 to December 31st 2021.

In the study population, 85.3% had a migration background, most commonly from Eastern EU countries. Fig 2 shows a detailed breakdown of the respective nationalities of participants in the study.

#### Seroprevalences of the four STI.

No acute HIV, HBV, or HCV infection was detected. Evidence of past HBV infection (anti‑HBc) was present in 14.6% (95% CI: 6.8–26.5). (7/48). One individual (2.1%; 95% CI: 0.2–9.3) had evidence of past HCV infection as a subsequent immunoblot test was positive, but below the detection limit of 30 IU/ml. Syphilis screening was reactive in 12.5% (6/48); two cases (4.2%; 95% CI: 0.9–12.7) met criteria for treatment‑requiring infection (positive IgM antibody test and a reactive lipoid antibody test), and four (8.3%; 95% CI: 2.9–18.6) reflected past infection. Co‑infections included one acute syphilis plus past HBV, and one past syphilis plus chronic HCV marker without viremia. Logistic regression (German or non-German and the sum of existing infectious diseases and chronic infections) indicated minimal explanatory value for citizenship status (Nagelkerke R² = 0.041). Vaccine‑induced immunity/Anti-HBs) was observed in 43.8% (21/48); 14/48 (29.2%) had titers ≥100 mIU/ml, indicating long‑term protection as per STIKO [21]. 37 of the 48 (77%) sex workers tested were healthy and suffered neither from an active STI nor were detectable as a chronic infection. An overview of the laboratory analyses can be seen in Table 3.

**Table 3:** STI serum tests and prevalence among sex workers in the PHS of Landshut by consultation year, January 01, 2019 to December 31, 2021.

		Number of positive/reactive test results (%)							
Years of investigation		2019		2020		2021		Gesamt	
Total number of examinations		29		11		8^§^		48	
Absolute and relative frequency distributions together with 95% confidence intervals		Frequency n (%)	95% CI n (%)	Frequency n (%)	95% CI n (%)	Frequency n (%)	95% CI n (%)	Frequency n (%)	95% CI n (%)
HIV^§^									
Chronic infection/acutely infected (%)	Antigen antibody screening test	0 (0.0)	–	0 (0.0)	–	0 (0.0)	–	0 (0,0)	–
Hepatitis B									
Vaccination coverage	anti-HBs	13 (44.8)	8-20 (27.9-62.7)	5 (45.5)	2-10 (20.0-73.0)	3 (37.5)	1-8 (11.9-70.5)	21 (43.8)	15-28 (30.4-57.8)
Chronic infection (%)	anti-HBc	4 (13.8)	1-9 (4.8-29.5)	2 (18.2)	0-6 (4.0-46.7)	1 (12.5)	0-4 (1.4-45.5)	7 (14.6)	3-13 (6.8-26.5)
Acutely infected (%)	HBs-Antigen	0 (0.0)	–	0 (0.0)	–	0 (0.0)	–	0 (0.0)	–
Hepatitis C									
	Antibody screening test	1 (3.4)	0-4 (0.4-15.0)	0 (0.0)	–	0 (0.0)	–	1 (2.1)	0-4 (0.2- 9.3)
Chronic infection (%)	Antibody confirmation test Bands^*)^	1 (100.0)	–	–	–	–	–	1 (2.1)	0-1
Acutely infected (%)	HCV-RNA- Verification^*)^	0 (0.0)	–	–	–	–	–	0 (0.0)	–
Syphilis									
	Antibody screening test (qualitative)	4 (13.8)	1-9 (4.8-29.5)	2 (18.2)	0-6 (4.0-46.7)	0 (0.0)	–	6 (12.5)	3-12 (5.4-24.0)
	IgM antibody confirmation test Bands^*)^	0 (0.0)	–	1 (50.0)	0-3 (6.1-93.9)	–	–	1 (16.7)	0-3 (1.9-55.8)
	Lipoid antibody detection (qualitative)^*)^	2 (50.0)	0-4 (12.3-87.7)	1 (50.0)	0-3 (6.1-93.3)	–	–	3 (50.0)	1-5 (16.7-83.3)
Chronic infection (%)		3 (10.3)	1-8 (3.0-25.1)	1 (9.1)	0-4 (1.0-35.3)	0 (0.0)	–	4 (8.3)	1-9 (2.9-18.6)
Acutely infected (%)		1 (3.4)	0-4 (0.4-15.0)	1 (9.1)	0-4 (1.0-35.3)	0 (0.0)	–	2 (4.2)	0-6 (0.9-12.7)

*) only carried out in the event of a positive screening test, § only 7 tests in 2021 (n = 47), – no tests and 95% CI due to negative screening tests

n = absolute frequencies.

%= relative frequencies.

#### Seroprevalences of acute STI: sex workers versus general population.

Against SurvStat@RKI data for Lower Bavaria [22] (NUTS 2; mean population ≈1.24 million), only two notifiable, treatment‑requiring syphilis cases were observed among the 48 serology participants across 2019–2021, with no acute HIV, HBV, or HCV cases (Table 4). Acute notifiable STI prevalence in this local sex worker cohort did not exceed that of the general population of the region.

**Table 4:** RKI reporting figures for acute HIV, hepatitis B/C and Syphilis in the Lower Bavaria (NB) territorial unit (source SurvStat@RKI 2.0, as of May 25 2022) and in the population of serum-tested sex workers in the Landshut public health department (SW_La_), January 01, 2019 to December 31, 2021.

Year of notification according to §7 IfSG	2019		2020		2021		Total	
Population	NB	SW_La_	NB	SW_La_	NB	SW_La_	NB	SW_La_
HIV	27	0	29	0	19	0	75	0
Hepatitis B	176	0	106	0	125	0	407	0
Hepatitis C	108	0	69	0	87	0	264	0
Syphilis	49	1	34	1	50	0	133	2
Total	360	1	238	1	281	0	879	2
Cumulative prevalence (%)	0.029	3.4	0.019	9.1	0.023	0.0		

## Discussion

This rural, monocentric pilot study provides initial estimates of STI prevalence and HBV immunization among registered sex workers. Contrary to assumptions of universally elevated risk, we observed no acute HIV, HBV, or HCV infections, arkers of past HBV infection were common and active/past syphilis was frequent. HBV immunization coverage—and especially long‑term protective titers—was suboptimal.

### Prevalence of defined STI

In contrast to data on HIV prevalence from major cities in Germany, the Netherlands and the German-Czech area (0.0–1.1%) during 2003–2008 [6–9,16,19], our pilot study did not reveal any HIV infections, which was somewhat unexpected. The higher prevalence reported in earlier studies likely resulted from PHS testing focused on high-risk populations (i.e., intravenous drug users) and presumably also included double counting of positive laboratory results [14], which may have inflated the prevalence estimates. Furthermore, it can be assumed that the low proportion of only 1.7% of male sex workers (men who have sex with men; MSM) as a potential high-risk group with the highest known group-specific HIV infection rates within Germany [23–25] represented a very low proportion within ourcohort. This low proportion of MSM in our cohort, but also the responsible preventive behavior of this group of people (i.e., use of condoms, group-specific educational measures, use of condoms) are unlikely to have contributed to any detectable HIV infections.

Although HBs antigen prevalence rates between 0.3–0.7% are reported for the German general population in studies published during the last few years [23–26]. Surprisingly no HBV infections (detection of HBs antigen) were detected within our study population, which contradicted the view of a generally increased risk of infection among sex workers [1,2]. Nevertheless, seven sex workers in the area of Landshut had previously been infected with HBV (anti-HBc), which statistically corresponded to a period prevalence of almost 15% in our laboratory analyses and was therefore very similar to the result of the Lübeck case-control study in cooperation with practicing gynecologists with an anti-HBc prevalence of 16.3% [7]. In contrast, the German-Czech “Jana” project from 1997–2001, which showed a greatly increased prevalence of HBs antigens of 4.5% [23], pointed in the opposite direction. This disparity in prevalence may be caused by completely different conditions (i.e., study design, study population, area of investigation) between our pilot study and the “Jana” project, therefore these results cannot be directly compared with our results.

Based on the literature, the vaccination coverage rate of 29% should be considered insufficient HBV protection, especially since a 2008 Lübeck study showed that only 6% of sex workers had protection against HBV [7].This great discrepancy between both studies could be interpreted as the Lübeck study been conducted well before the introduction of ProstSchG and the resulting sensitization to vaccinations, meaning possible preventive effects wre not yet in effect at that time.. Further, a cross-sectional study conducted in Vancouver in 2019 showed that 68.3% of the sex workers had received at least one dose of HBV vaccine [4]. This Canadian study also showed that migrants had a significantly lower chance of being vaccinated (adjusted OR=0.5; 95% CI:0.3–0.8) [4], although this was not confirmed in our cohort. One possible reason for the comparatively high vaccination coverage rate in this Canadian study may have been primarily due to the study design with self-reported questionnaires, which may have contributed to such high vaccination coverage rates in the sense of overreporting due to recall or social desirability bias.

In opposite findings align with selected local and international reports showing heterogeneity by setting, sample composition, and testing strategy [4–9,16,17,19,22,23]. Differences from urban studies likely reflect local epidemiology, service access, and the voluntary nature of testing. The relatively high proportion of sex workers with a migration background underscores culturally and linguistically appropriate services.

### Sexworkers versus general population

When comparing the prevalence of STIs between our local study area and the overall population of Lower Bavaria, it must be critically considered that our 48 positive STI laboratory findings represent a very low prevalence compared to the overall Lower Bavarian population of approximately 1.3 million. Certainly, it should be noted that the reported prevalence for Lower Bavaria included men and women of all age groups in a highly aggregated form, but our study population had not such socio-epidemiological structure. Due to the voluntary nature of STI testing, the potential for infection in the population of sex workers can only be made available on a random basis and therefore not representatively for the entire population of registered sex workers. This means that personal responsibility and the right to self-determination take priority in these voluntary serum analyses, where the focus has been on monitoring the prevalence of infection and not on monitoring it among registered sex workers [10]. However, the proportion of undeclared sex workers (e.g., those who engage in this activity occasionally, etc.) should not be disregarded and it can be assumed that this group of registered sex workers may well be at a higher risk of STIs than legal sex workers.

### Strengths and limitations of the study

Strengths include direct access to a defined registered workforce, standardized routine data, and confirmatory laboratory testing. Limitations include small sample size, possible selection bias due to voluntary testing, limited temporal resolution, lack of detailed behavioral risk data, and restricted generalizability beyond registered workers. These factors support interpreting estimates as exploratory and hypothesis‑generating.

## Conclusions

In this rural German setting, acute notifiable STIs were uncommon among registered sex workers; syphilis and past HBV infection were more prominent, and HBV immunization coverage was insufficient. Tailored vaccination and syphilis prevention programs in PHS are warrented, alongside further research in larger, multi‑site cohorts.

From a public health perspective, the priority areas are: (i) enhancing HBV vaccination coverage and completion; (ii) strengthening syphilis screening/treatment and partner notification; (iii) maintaining low‑threshold, trust‑based services integrated with counseling mandated by ProstSchG; and (iv) monitoring trends through consistent, non‑stigmatizing surveillance.
